# Red Cabbage Rather Than Green Cabbage Increases Stress Resistance and Extends the Lifespan of *Caenorhabditis elegans*

**DOI:** 10.3390/antiox10060930

**Published:** 2021-06-08

**Authors:** Nan Zhang, Shunshan Jiao, Pu Jing

**Affiliations:** Shanghai Food Safety and Engineering Technology Research Center, Key Lab of Urban Agriculture Ministry of Agriculture, School of Agriculture & Biology, Shanghai Jiao Tong University, Shanghai 200240, China; nzhang@sjtu.edu.cn (N.Z.); sjiao@sjtu.edu.cn (S.J.)

**Keywords:** lifespan, anthocyanins, phytochemicals, HSP-1 pathway, CaMKII pathway, HSP-1 pathway, caco-2 cells

## Abstract

Many studies have demonstrated that cabbages possess various biological activities, and our previous studies confirmed that cyanidin-3-diglucoside-5-glucoside (CY3D5G), the major core of red cabbage anthocyanins, exhibited in vitro antioxidant activity. This study further investigated the protective effects of CY3D5G derivative from red cabbage juice (RCJ) on oxidative stress and lifespan in cells and *Caenorhabditis elegans*, green cabbage juice (GCJ) was used as control. RCJ rather than GCJ significantly improved cell viability and decreased lactate dehydrogenase release in H_2_O_2_-induced caco-2 cells. RCJ significantly increased survival during oxidative and heat stress and mean lifespan in *C. elegans* by 171.63% and 31.64%, and 28.16%, respectively, while GCJ treatment showed no significant effects (*p* < 0.05). These results might be attributed to significantly (*p* < 0.05) higher contents of total phenolics, ascorbic acid, glucosinolates, and anthocyanins in RCJ compared to those in GCJ. Additionally, both of them decreased autofluorescence and reproductive capacity, increased body length, but did not alter the intracellular ROS level. Prolonged lifespan by RCJ might require heat-shock transcription factor pathway, sirtuin signaling, and calmodulin kinase II pathway, independent of insulin/insulin-like growth factor-1 signaling pathway. RCJ showed promising antioxidant properties in caco-2 cells and *C. elegans*, which provided more information on the health benefits of cabbage.

## 1. Introduction

Aging is a universal and inevitable biological phenomenon. According to the free radical theory of aging, oxidative stress damages various cell components and activates specific signal pathways, which influences numerous cellular processes linked to aging [1]. Supplementation of antioxidants has been provided to reduce the oxidative stress level in model organisms [1]. The optimal source of antioxidants might come from diet. Diet has significantly impact on physiological well-being, and high intake of vegetables has been proved to be beneficial in aging and age-related diseases [2].

Cabbage, which belongs to the family of Cruciferae, is one of the most important dietary vegetables consumed in Asia, such as red cabbage (*Brassica oleracea* L. var. capitata L. f. rubra) and green cabbage (*Brassica oleracea* L. var. capitata L.). One hundred grams of cabbage contains 5.8 g of carbohydrate, 1.28 g of protein, 0.1 g of fat, 2.5 g of fiber, 40 mg of calcium, and 0.47 mg of iron. Additionally, cabbage contains numerous phytochemicals such as phenolics, glucosinolates, vitamins, and anthocyanins [3]. Recent studies indicated that the extracts from cabbage possess a range of biological activities, including cardioprotective, hepatoprotective, hypoglycemic, and hypolipidemic effects [4,5,6]. Our previous study investigated the in vitro antioxidant effects of cyanidin-3-diglucoside-5-glucoside (CY3D5G) from red cabbage. Results suggested that CY3D5G relieved cellular reactive oxygen species (ROS) level and exhibited antioxidant activities in H_2_O_2_-induced RAW264.7 cells via Nrf2/HO-1 signaling pathways [7,8]. However, the effects of CY3D5G derivative from red cabbage in cells and a whole organism have not been fully characterized.

*Caenorhabditis elegans* is a widely accepted and established model organism with the advantages of ease of culture, relatively short lifespan, a simple structure, and a distinct molecular signaling pathway [9]. *C. elegans* contains more than 18,000 genes where 60–80% consist of human gene homologs [10]. These conserved pathways include insulin/insulin-like growth factor (IGF)-1 signaling (IIS) pathway, calmodulin kinase II (CaMKII) pathway, and heat-shock transcription factor (HSF-1) pathway, which are vital for stress resistance and lifespan regulation [11]. *C. elegans* has been widely used to study anti-aging activity in recent years. Chen et al. [12] reported that the extract of 3 Tsai Tai (*Brassica chinensis*) varieties, cruciferous vegetables, significantly decreased the intracellular ROS level, and the extract of Hon Tsai Tai extend lifespan in *C. elegans*.

The aims of the present research were to understand i) if red cabbage juice (RCJ) containing unique phytochemicals rather than green cabbage juice (GCJ) extends the lifespan of *C. elegans*, and ii) the relationship of oxidative stress of *C. elegans* with its lifespan and healthspan. For those purposes, we evaluated the antioxidant and anti-aging actions of RCJ and GCJ with distinctive phytochemicals in models of H_2_O_2_-induced caco-2 cells and *C. elegans*. The mechanisms were also investigated using *C. elegans* strains N2, GR1307 [*daf-16(mgDf50)*I], VC199 [*sir-2.1(ok434)*IV], and MT2605 [*unc-43(n498n1186)*IV].

## 2. Materials and Methods

### 2.1. Materials

Folin–Ciocâlteu reagents were purchased from Hushi Laboratory Equipment Co. (Shanghai, China). 2′,7′-Dichlorofluorescin diacetate (DCFH-DA), sinigrin (purity ≥ 99%), and TRI reagent were obtained from Sigma-Aldrich (Shanghai, China). Ascorbic acid (purity ≥ 99%), 5-fluoro-2-deoxyuridine (5-FUDR), ampicillin, and paraquat were purchased from Aladdin Chemical Reagents Co. (Shanghai, China). Dulbecco’s modified Eagle’s medium (DMEM) high glucose medium was obtained from KeyGEN BioTECH (Shanghai, China). Fetal bovine serum (FBS) was purchased from Gibco (Gibco, Waltham, MA, USA). Lactate dehydrogenase (LDH) assay kit and CCK-8 cell viability assay kit were purchased from Nanjing Jiancheng Bioengineering Institute (Nanjing, China). PrimeScript^TM^ RT reagent Kit and SYBR^®^ Premix Ex Taq™ were provided by Takara Biotechnology (Shiga, Japan). The oligonucleotide primers were synthesized from Shanghai Generay Biotech Co., Ltd. (Shanghai, China). All other chemicals were purchased from Hushi Laboratory Equipment Co. (Shanghai, China).

### 2.2. Preparation of RCJ and GCJ

The red and green cabbage heads were purchased from a local market in Shanghai, China, in September 2019. Three heads of red cabbage or green cabbage were sliced into pieces and squeezed to juice by a low-speed juicer (MIUIB03C, Xiaomi, China). The juice was centrifuged at 10,000× *g* for 10 min at 4 °C. The supernatant was collected and stored at −20 °C for further analysis.

### 2.3. Determination of Total Phenolics

Total phenolics were determined using a modified Folin–Ciocâlteu method as described by Zhang et al. [13]. Briefly, 0.5 mL of samples was mixed with 2.5 mL Folin–Ciocâlteu reagents (diluted 1:10 with distilled water). After that, 2 mL of 75 g/L Na_2_CO_3_ solution was added and mixed before incubation at 37 °C for 15 min. Then, the absorbance was measured at 760 nm by UV–vis spectrophotometer (BIOMATE 3S, Thermo Fisher Scientific, Waltham, MA, USA). The results were calculated using a standard curve of gallic acid and expressed as mg gallic acid equivalents (GAE)/100 mL of juice.

### 2.4. Determination of Ascorbic Acid

Ascorbic acid was determined by high-performance liquid chromatography (HPLC) as described by Denardin et al. [14]. A volume of 1 mL of juice was mixed with 2 mL of extraction solution containing 8% acetic acid (*v*/*v*) and 3% meta-phosphoric acid (*v*/*v*) and allowed to extract for 30 min. The mixture was centrifuged at 12,000× *g* for 10 min at 4 °C. The supernatant was collected and filtered through 0.22 μm filters (Tansoole, Shanghai, China) for HPLC analysis. Ascorbic acid was determined using a Shimadzu LC-2030C HPLC (Shimadzu, Kyoto, Japan) system. Separation was obtained by reverse phase elution on a C18-WP column (1.7 μm, 100 mm × 2.1 mm, ANPEL, Shanghai, China). Mobile phase was 0.01% sulfuric acid-water for isocratic elution. An injection volume of 10 μL with 1 mL/min flow rate was used. The UV detection wavelength was 260 nm. Identification was achieved by comparing retention time with standard. Ascorbic acid was quantified by external calibration and expressed as mg/100 mL of juice.

### 2.5. Determination of Glucosinolates

A modified HPLC method was used to determine the total glucosinolates content [15]. Briefly, 5 mL volumes of RCJ and GCJ were mixed with 25 mL of methanol and immersed in a water bath at 75 °C for 10 min. The mixtures were centrifuged at 2000× *g* for 10 min at 4 °C with the addition of 5 mL of barium acetate (0.4 mol/L) and supernatants were collected. Residual methanol in the supernatant was removed in a rotary evaporator. Distilled water was added to bring the total volume to 10 mL. A 5 mL aliquot of extract was loaded onto a DEAE-Sephadex A-25 and washed with 5 mL of distilled water. Aryl sulphatase (100 μL) was loaded onto a column. After an overnight incubation period, the desulphonated glucosinolates were eluted with 3 mL of distilled water and refrigerated at 12,000× *g* for 10 min. The supernatant was collected and filtered through 0.22 μm filters (Tansoole, Shanghai, China) for HPLC analysis. The desulpho-glucosinolates was analyzed using a Shimadzu LC-2030C HPLC (Shimadzu, Kyoto, Japan) system. Separation was obtained by reverse phase elution on a Shim-pack VP-ODS column (5 μm, 250 mm × 4.6 mm, Shimadzu, Kyoto, Japan) fitted with a 4.6 × 10 mm Shim-pack GVP-ODS guard column (Shimadzu, Kyoto, Japan). Mobile phases were: (A) 0.1% formic acid-water and (B) acetonitrile. Separation was achieved through a gradient elution: 0–10 min, 10% B; 10–15 min, 10–25% B; 15–35 min, 25% B; 35–40 min, 25–10% B. An injection volume of 20 μL with 1 mL/min flow rate was used. The UV detection wavelength was 229 nm. Identification was achieved by comparing retention time with standard. Total amount of glucosinolates was calculated as sinigrin equivalent and expressed as mg sinigrin equivalent/100 mL of juice.

### 2.6. Determination of Total Anthocyanin

The total anthocyanins of RCJ were determined using pH differential method described previously [7]. Briefly, RCJ was diluted using KCl solution (0.025 mol/L, pH 1.0) and NaAc buffers (0.4 mol/L, pH 4.5). The solution was allowed to stay for 1 h in the dark. Absorbance was determined at 530 and 700 nm using the UV–vis spectrophotometer (BIOMATE 3S, Thermo Fisher Scientific, USA). Total anthocyanins were calculated as:

(1) $$\mathrm{Total}=\frac{(\left(A_{530}-A_{700}{)}_{pH\;1}-{\left(A_{530}-A_{700}\right)}_{pH\;4.5}\right)\times \mathrm{DF}\times 1000\times \mathrm{MW}}{\varepsilon }$$

where total anthocyanins were expressed as cyanidin 3-glucoside (C3G) with a molar absorptivity (ε) of 26,900 × cm^−1^ mg^−1^ and a molecular weight (MW) of 449.2. DF was the dilution factor. The total anthocyanins content was expressed as mg cyanidin-3-glucoside equivalent/100 mL of RCJ.

### 2.7. Extraction and HPLC-MS Analysis of Anthocyanins

Anthocyanins were extracted and identified according to previously reported procedures [7]. A volume of 1 mL of juice was mixed with 3 mL of 0.1% hydrochloric acid-methanol and the mixture was centrifuged at 12,000× *g* for 10 min at 4 °C. The supernatant was collected and filtered through 0.22 μm filters (Tansoole, Shanghai, China). The anthocyanins in RCJ were identified using Acquity I-class ultra-performance liquid chromatography (Acquity UPLC™) system (Waters Corporation, Milford, MA, USA) coupled with a Vion IMS Q-TOF mass spectrometer (Waters Corporation, Milford, MA, USA). Separation was obtained on a BEH C18 column (1.7 μm, 100 mm × 2.1 mm i.d., Waters, Milford, MA, USA). The mobile phases were: (A) 0.1% formic acid-water and (B) 0.1% formic acid-acetonitrile. A gradient program was used for mobile phases as follows: 0–3 min, 5–20% B; 3–10 min, 20–100% B; 10–12 min, 100% B; 12–15 min, 100–95%; 15–19 min, 95%. An injection volume of 1 μL with 0.4 mL/min flow rate was used. Data were collected at 530 nm. The applied electrospray/ion optic parameters were set according to previous studies: Capillary voltage, 2.0 kV; cone voltage, 40 V; desolvation temperature, 450 °C; desolvation gas, 900 L/h; cone gas, 50 L/h; source temp, 115 °C; scan rate, 0.2 s; collision energy, 6 eV. Spectra were collected using MS^E^ scan mode over the mass-to-charge (*m*/*z*) ratio range of 50–1000 au in positive mode. Data were collected and analyzed with UNIFI software (v 18.2.0, Waters Corp., Milford, MA, USA). Identification of each anthocyanin was achieved by comparing molecular weight and fragmentation patterns to those reported in available references.

### 2.8. Cell Culture and Viability Assay

Caco-2 cells were obtained from Chinese cell bank (Shanghai, China) and cultured in DMEM high glucose medium supplemented 10% FBS at 37 °C humidified incubator containing 5% CO_2_.

Cell viability was tested by CCK-8 assay according to a previous method [7]. Briefly, caco-2 cells were plated at a density of 3 × 10^4^ cells/well into a 96-well microplate and allowed to grow for 24 h. Then, cells were treated with 1–5% of RCJ and GCJ for 24 h. CCK-8 reagent was added and incubated for 1 h at 37 °C in a 5% CO_2_ humid atmosphere. The reduction in CCK-8 was quantified by Infinite F200 PRO microplate reader (Tecan, Switzerland) at 450 nm. The cell viability was calculated as a relative percentage of control.

### 2.9. RCJ and GCJ Pre-Treatment and H_2_O_2_-Induced Oxidative Injury Model

Caco-2 cells were seeded into a 96-well microplate at a density of 3 × 10^4^ cells/well for 24 h. Then, the cells were incubated with 1%, 2%, 3%, and 5% of RCJ and GCJ for 24 h, respectively. After 24 h incubation, the treatment medium was removed and treated with 1 μM of H_2_O_2_ for 2 h. The cell viability was determined using the method shown in Section 2.8.

### 2.10. Assay of LDH in H_2_O_2_-Induced Caco-2 Cells

The activity of lactate dehydrogenase (LDH) in the supernatants was determined using commercial assay kit (Nanjing Jiancheng Bioengineering Institute, China). Caco-2 cells were seeded into a 96-well plate at the density of 3 × 10^4^ cells/well and allowed to grow for 24 h. Cells were then pre-treated with RCJ and GCJ for 24 h and later stimulated with 1 mM of H_2_O_2_ for 2 h. The supernatant was collected and assayed for LDH following the manufacturer’s protocols.

### 2.11. Strains and Growth Conditions

*C. elegans* strains N2 (wild-type), GR1307 [*daf-16(mgDf50)*I], VC199 [*sir-2.1(ok434)*IV], and MT2605 [*unc-43(n498n1186)*IV] were obtained from the *Caenorhabditis* Genetics Center (University of Minnesota, Minneapolis, MN). All worm strains were maintained at 20 °C on solid nematode growth medium (NGM) plated with *Escherichia coli* OP50 as food source [16]. Synchronized worms were obtained by allowing about 10 hermaphrodites to lay eggs for 4 h before removing them.

### 2.12. Lifespan Assay

Lifespan was monitored as described previously [17]. On day 0 of the experiment, synchronized L4 larvae worms were transferred to NGM plates containing 1%, 2%, 3%, and 5% of either RCJ or GCJ, 50 μM of 5-FUDR to prevent the growth of progeny, and 100 µg/mL of ampicillin to arrest bacterial growth. Worms were transferred to fresh medium and scored for survival every other day. Worms were judged to be dead when they did not respond to a gentle touch with the platinum wire. Worms with internal hatching and missing worms were censored. Kaplan–Meier lifespan analysis was carried out, and *p* values were calculated using the log-rank test, *p* < 0.05 was accepted as statistically significant. All statistical analyses were performed using SPSS software (version16.0, SPSS Inc., Chicago, IL, USA).

### 2.13. Stress Resistance Assay

To evaluate stress resistance, synchronized wild-type worms at L1 larval stage were treated with RCJ and GCJ for 3 days followed by exposure to the stressor. For the oxidative stress resistance assay, animals were exposed to 10 mM paraquat and survival rates were scored at 3.5 days. For the heat stress resistance assay, animals were incubated at 35 °C for 4 h, and then plates were removed from the incubator and scored for survival. The assay was repeated three times with *n* > 60 worms per group.

### 2.14. Autofluorescence

Briefly, synchronized L4 larvae worms (*n* = 20 per group) were treated with different concentrations of RCJ and GCJ for 8 days. Worms were mounted on 2% agarose pads on a glass slide and immobilized with 0.25 μM levamisole. Slides were visualized using Leica inverted microscope DMi8 (Leica Microsystems, Wetzlar, Germany) equipped with a DAPI filter set. The bright field and fluorescence images were captured with a Leica DFC365 FX camera using Leica Application Suite X. The images were taken at 100× magnification. The fluorescence intensity was measured using Image J software and normalized to the body size [18].

### 2.15. Quantification of Intracellular ROS

Synchronized worms at L1 larval stage were cultivated on NGM with different concentrations of RCJ and GCJ for 48 h. Subsequently, 30 worms were collected and transferred to a 96-well plate containing H_2_DCF-DA (final concentration was 25 μM). The fluorescence intensity was recorded every 10 min for 4 h in an Infinite F200 Pro microplate reader (Tecan, Männedorf, Switzerland) with 485 nm excitation and 530 nm emission [19].

### 2.16. Body Length and Brood Size Assays

Body length and brood size assays were performed essentially as previously described [20]. Briefly, synchronized L4 larvae were treated with different concentrations of RCJ and GCJ for 5 days. Images were captured and the body length was measured along the animal axis using Image J software. To assay the brood size, synchronized L4 larvae worms were transferred to control plates or treatment plates with different concentrations of RCJ and GCJ with one worm per plate (*n* = 10 per group). Worms were transferred to new plates every day until the end of the reproductive period. The total number of the offspring that grew up from each animal was counted.

### 2.17. Real-Time Quantitative Polymerase Chain Reaction (RT-qPCR)

The worms at L1 larval stage were grown on NGM plates with or without 5% RCJ for 2 days. Then, the worms were collected and washed three times in M9 buffer. Total RNA was isolated from approximately 2000 worms in each group using TRI reagent, and cDNA was synthesized using the PrimeScript™ RT reagent Kit according to the manufacturer’s protocol. RT-qPCR was performed using SYBR^®^ Premix Ex Taq™ by qTower 3G real-time PCR system (Analytik Jena AG, Jena, Germany). Primer sequences, genes accession number, annealing temperature, and primer pair efficiency are shown in Supporting Information Table S1. The qPCR amplifications were performed with initial denaturation at 95 °C for 30 s, followed by 40 cycles of 95 °C for 3 s and 60 °C for 30 s. The formation of PCR products was confirmed by melting curve analysis. The 2^−ΔΔCt^ method was used to calculate the relative mRNA expression levels. The *act-1* was used as an internal control for normalization. Each group was measured in six biological replicates, and each biological replicate was measured in technical triplicates.

### 2.18. Data Analysis

Statistical analysis of data was performed by one-way ANOVA followed by Bonferroni correction using SPSS (version16.0, SPSS Inc., Chicago, IL, USA). Tests were conducted in triplicate determinations with data reported as means ± standard deviation (SD).

## 3. Results

### 3.1. Total Phenolics, Ascorbic Acid, Glucosinolates, and Anthocyanins Contents in RCJ and GCJ and Identification of Anthocyanins in RCJ

Phenolics, ascorbic acid, glucosinolates, and anthocyanins contents of RCJ and GCJ are shown in Table 1. The total phenolics were 29.95 ± 0.94 and 17.20 ± 0.15 mg GAE/100 mL of juice in RCJ and GCJ, respectively. Ascorbic acid content of RCJ was 30.55 ± 0.03 mg/100 mL of juice, which was significantly (*p* < 0.05) higher than that of GCJ (21.68 ± 0.17 mg/100 mL of juice). RCJ presented higher glucosinolates content (70.21 ± 3.64 mg sinigrin equivalent/100 mL of juice) as compared to GCJ (59.66 ± 1.04 mg sinigrin equivalent/100 mL of juice). Additionally, total anthocyanin was 33.87 ± 0.60 mg/100 mL of juice expressed as C3G equivalents in RCJ.

### 3.2. Identification of Anthocyanins in RCJ

**Figure 1:**
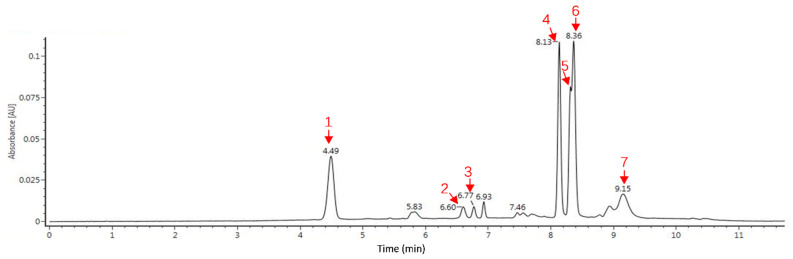
UPLC profile of anthocyanins in red cabbage juice. The corresponding compounds are list in Table 2.

The main anthocyanins were identified by ultra-performance liquid chromatography-mass spectrometry (UPLC-MS), and the UPLC profiles are presented in Figure 1. Altogether, seven anthocyanins were identified in our study (Table 2), and all of them were based on a core of cyanidin 3-diglucoside-5-glucoside. The most prevalent anthocyanins detected in RCJ were acylated with caffeic, *p*-coumaric, ferulic, and/or sinapic acids.

### 3.3. RCJ exhibited Antioxidant Activity in Caco-2 Cells

Caco-2 cells, similar to the intestinal epithelial cells, partly reflect the intestinal absorption and are widely used to reflect potential antioxidant activity. The CCK-8 assay was carried out to determine the cytotoxicity of RCJ and GCJ. According to the result, the cell viabilities for all treatment groups were higher than 80%, indicating that no cytotoxic effects were observed.

**Figure 2:**
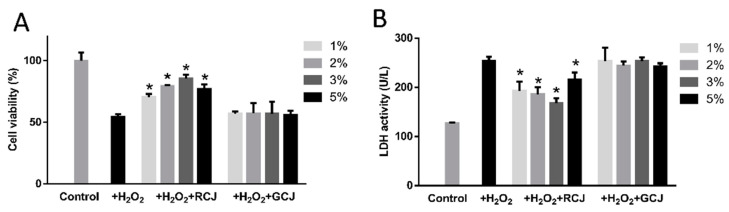
Effect of red cabbage juice (RCJ) and green cabbage juice (GCJ) on the cell viability (**A**) and lactate dehydrogenase (LDH) release (**B**) in H_2_O_2_-induced caco-2 cells. Data are presented as mean ± SD. * *p* < 0.05 vs. control by one-way ANOVA followed by Bonferroni correction.

As shown in Figure 2A, cell viability decreased to 54.60% after being exposed to 1 mM of H_2_O_2_ for 2 h, suggesting that a model of H_2_O_2_-induced injury in caco-2 cells was successfully established. RCJ treatment significantly improved the cell viability (*p* < 0.05), while GCJ showed no effects compared with the model group (*p* > 0.05). The LDH activity in the control group significantly increased to 253.42 U/L compared to the model group (127.25 U/L) (*p* < 0.05). RCJ treatment groups, which were pre-incubated with 1–5% RCJ, significantly decreased LDH activity to 192.73 U/L (1% RCJ), 185.87% (2% RCJ), 167.78% (3% RCJ), and 215.98 (5% RCJ) U/L, respectively (Figure 2B, *p* < 0.05). In contrast, treatment with GCJ had no impact on the release of LDH.

### 3.4. RCJ enhanced the Oxidative and Thermal Stress Resistance

**Figure 3:**
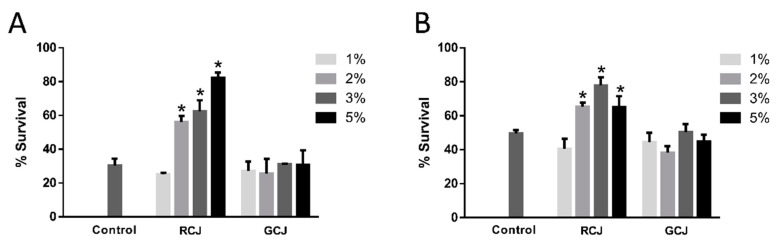
Effect of pretreatment with red cabbage juice (RCJ) and green cabbage juice (GCJ) on resistance to stress in *C. elegans*. Synchronized L1 larvae worms were treated with RCJ and GCJ for 3 days and exposed to stressors: (**A**) 10 mM paraquat for 3.5 days (*n* > 60); (**B**) 35 °C heat shock for 4 h (*n* > 60). Data are presented as mean ± SD. * *p* < 0.05 vs. control by one-way ANOVA followed by Bonferroni correction.

In addition to in vitro antioxidant activities, the in vivo antioxidant capacities of RCJ and GCJ were also investigated in *C. elegans*. Wild-type N2 worms were pretreated with and without RCJ and GCJ for 3 days and then exposed to paraquat and heat. After treatment with 10 mM of paraquat for 3.5 days, approximately 70% of the worms had died. At that time, 56.11%, 62.52%, and 82.25% of worms treated with 2%, 3%, and 5% RCJ were alive, respectively (Figure 3A), suggesting that RCJ supplementation enhanced resistance to oxidative stress in *C. elegans*. However, 1–5% GCJ treatments did not increase the survival rate of wild-type worms under oxidative stress. A similar phenomenon was observed in the heat tolerance conditions (Figure 3B). Treatment with 5% RCJ led to 171.63% and 31.64% increase in the survival rate under oxidative conditions and high temperature, respectively.

### 3.5. RCJ and GCJ Attenuated Autofluorescence Accumulation but Do Not Affected ROS Level

**Figure 4:**
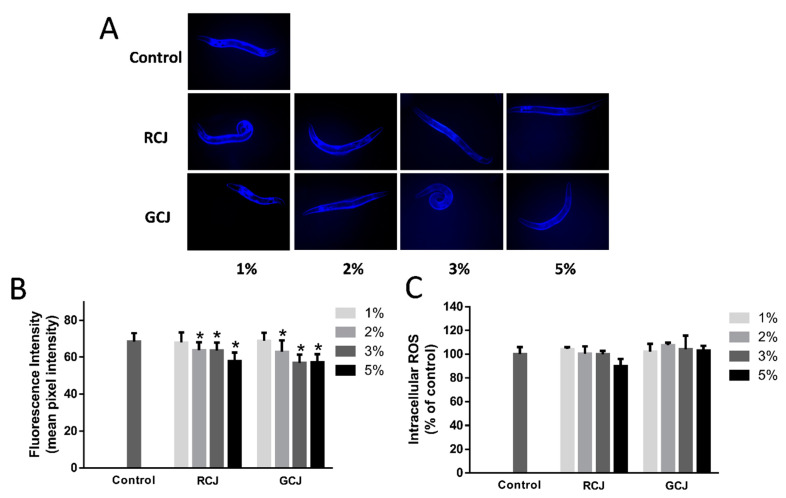
Effect of red cabbage juice (RCJ) and green cabbage juice (GCJ) on autofluorescence (**A**,**B**) and ROS (**C**) accumulation. (**A**) Representative images of autofluorescence from RCJ- and GCJ-treated worms on day 8. The images were taken with a DAPI filter set and at 100× magnification. (**B**) Mean fluorescence intensity from autofluorescence in day 8 worms treated with RCJ and GCJ (*n* = 20). (C) Synchronized L1 larvae worms were treated with RCJ and GCJ for 2 days and collected for determination of ROS level using DCF assay (*n* = 30). Data are presented as mean ± SD. * *p* < 0.05 vs. control by one-way ANOVA followed by Bonferroni correction.

Autofluorescence is a byproduct of lysosomal degradation which accumulated in aging cells. Wild-type N2 worms treated with 2%, 3%, and 5% RCJ and GCJ for 8 days significantly (*p* < 0.05) decreased the accumulation of autofluorescence (Figure 4A). In the fluorescence photography assays, intestinal autofluorescence levels were reduced by 18.19% and 19.79% in 5% RCJ and GCJ-treated worms compared with control, respectively (Figure 4B). The effects of RCJ and GCJ on the intracellular ROS were measured using H_2_DCF-DA. After treatment with RCJ and GCJ for 2 days, no significant differences (*p* > 0.05) were observed between RCJ and GCJ treatment groups and the control (Figure 4C).

### 3.6. RCJ and GCJ Decreased Brood Size and Increased Body Length

**Figure 5:**
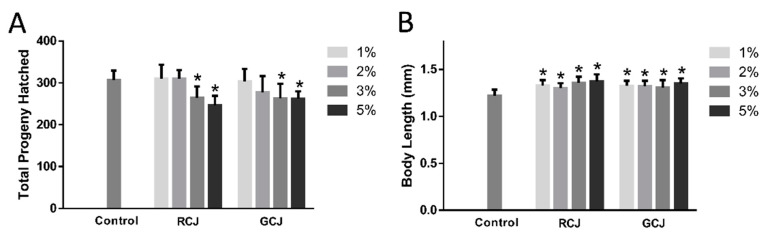
Effect of red cabbage juice (RCJ) and green cabbage juice (GCJ) on brood size (**A**) and body length (**B**) of *C. elegans*. (**A**) Synchronized L4 larvae were individually transferred to a fresh plate each day until reproduction ceased (*n* = 10). (**B**) Synchronized L4 larvae worms were treated with RCJ and GCJ. Photographs were taken of animals on day 5, and the body length of each individual nematode was analyzed by Image J software (*n* = 30). The images were taken at 100× magnification. Data are presented as mean ± SD. * *p* < 0.05 vs. control by one-way ANOVA followed by Bonferroni correction.

Brood size and body length of worms were tested to reflect the general fitness after RCJ and GCJ treatment. RCJ and GCJ treatments significantly (*p* < 0.05) decreased the total offspring quantity compared to control in a dose-dependent manner (Figure 5A). Body length was determined in wild-type worms treated with or without RCJ and GCJ for 5 days. As shown in Figure 5B, the body length for worms treated with 1%, 2%, 3%, and 5% RCJ was significantly increased by 8.72%, 6.63%, 10.95%, and 12.52%, respectively. Similarly, 1%, 2%, 3%, and 5% GCJ increased body length by 8.41%, 8.26%, 6.98%, and 10.42%, respectively.

### 3.7. RCJ Extend the Lifespan of Wild-Type C. elegans

**Figure 6:**
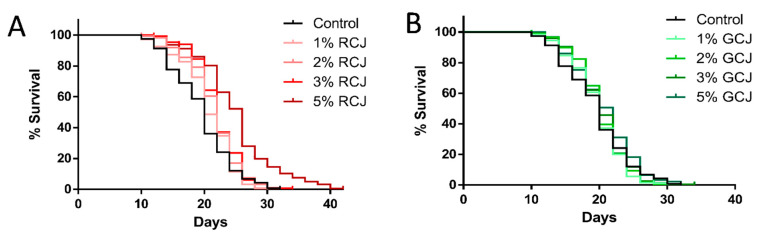
Effect of red cabbage juice (RCJ) and green cabbage juice (GCJ) on the lifespan of wild-type *C. elegans*. Synchronized L4 larvae worms were treated with or without 1%, 2%, 3%, and 5% RCJ (**A**) and GCJ (**B**). Three independent trials were performed.

We further investigated whether RCJ and GCJ treatment could affect the lifespan of *C. elegans* under standard laboratory conditions. Four concentrations of RCJ and GCJ range from 1 to 5% were tested. As shown in Figure 6A, worms treated with 2% and 3% RCJ displayed a significant right-shift survival curve compared with the control, and the survival curve of 5% RCJ was continuously above that of control. RCJ at 2%, 3%, and 5% significantly (*p* < 0.05, by the log-rank test) extended the lifespan by 10.37%, 13.04%, and 28.18% in comparison to the control, respectively (Table 3). Wild-type worms treated with GCJ did not show a significant increase in lifespan (Figure 6B), but GCJ treatment exhibited a slight increase in mean lifespan (Table 3). The mean lifespan of worms treated with 1%, 2%, 3%, and 5% RCJ significantly increased by 5.25%, 7.52%, 9.18%, and 21.45%, respectively, as compared to GCJ at the same concentration.

### 3.8. Genetic Requirements for Increased Survival from RCJ Treatment

RT-qPCR was performed to analyze the transcriptional level of some key genes to explore how RCJ prolongs the lifespan of *C. elegans*. Whether mutations in three major stress response and longevity pathways, e.g., *daf-16(mgDf50)*, *sir-2.1(ok434)*, and *unc-43(n498n1186)* mutant, impaired the ability of RCJ to prolong lifespan was investigated.

**Figure 7:**
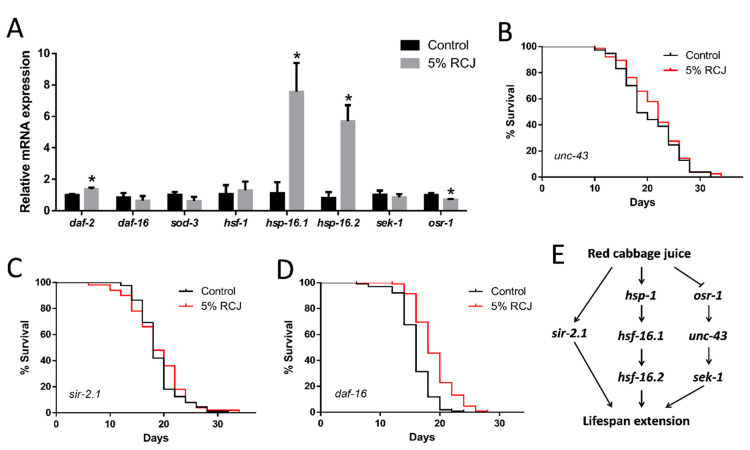
The molecular mechanism of red cabbage juice (RCJ) in lifespan extension. (**A**) Relative mRNA level of *daf-2*, *daf-16*, *sod-3*, *hsf-1*, *hsp-16.1*, *hsp-16.2*, *sek-1*, *osr-1* after 2 days of 5% RCJ treatment. Data are presented as mean ± SD. * *p* < 0.05 vs. control by one-way ANOVA followed by Bonferroni correction. (**B**) Survival curves of *daf-16(mgDf50)*. (**C**) Survival curves of *sir-2.1(ok434)*. (**D**) Survival curves of *unc-43(n498n1186)*. (**E**) Hypothetical model of action of the longevity extension mediated by RCJ.

IIS is known to regulate aging and longevity. As shown in Figure 7A, RCJ up-regulated the mRNA level of *daf-2*, while *daf-16* and *sod-3* mRNA levels were not altered in wild-type worms. The *daf-16(mgDf50)* mutant treated with 5% RCJ displayed a significantly (*p* < 0.05 by the log-rank test) extended mean lifespan compared to the control (Figure 7B). These results suggested that RCJ might act independently of IIS pathway.

Heat shock factor 1 (HSF-1) is another transcription factor crucial for longevity downstream of IIS. As shown in Figure 7A, RCJ treatment markedly increased the mRNA expression of *hsp-16.1* and *hsp-16.2* (*p* < 0.05), indicating that the HSF-1 pathway might be involved in the mechanism of lifespan extension. We hypothesized that RCJ might go through the HSF-1 pathway to improve stress resistance. Additionally, RCJ treatment did not extend the lifespan of *sir-2.1(ok434)* worms (*p* < 0.05 by the log-rank test; Figure 7C), indicating that *sir-2.1* was required for the longevity effect of RCJ.

Whether the protective effect of RCJ through OSR-1/UNC-43/SEK-1 stress response pathway was investigated. As shown in Figure 7D, RCJ treatment did not prolong lifespan of *unc-43(n498n1186)* worms, suggesting that RCJ might act through *unc-43*. Additionally, RCJ treatment significantly down-regulated the transcription level of *osr-1* (*p* < 0.05), but did not alter *sek-1* mRNA level (Figure 7A). These results implied that the OSR-1/UNC-43 pathway was a target for RCJ in *C. elegans*.

## 4. Discussion

Fruits and vegetables have been recommended worldwide in dietary guidance since the abundant phytochemicals and vitamins [21]. Red and green cabbages are fresh edible vegetables, which contain amounts of bioactive compounds. The contents of total phenolics, ascorbic acid, and glucosinolates in RCJ were 74.13%, 40.91%, and 17.68% higher than those of GCJ, respectively, which was consistent with previous studies [3,22]. Red cabbage anthocyanins are typically cyanidin mono- or di-acylated with hydroxycinnamic acids [23]. Mizgier et al. [24] reported a total of 21 anthocyanins in red cabbage extracted with acid acetone.

H_2_O_2_ is continually generated during normal metabolism and has been used to construct cell oxidative injury model [25]. CCK-8 assay measured the redox activity of living cells. LDH releases into the medium from dead cells, which is a good indicator of cellular damage. RCJ treatment significantly improved the cell viability and decreased the cell death in H_2_O_2_-induced oxidative injury model. Similarly, RCJ greatly improved survival following paraquat-induced oxidative stress and heat shock in *C. elegans*. GCJ exhibited no protective effect against H_2_O_2_-induced oxidative stress, nor did it show increased resistance to oxidative and heat stress in *C. elegans*. Phytochemicals-rich extracts have been shown to exhibit antioxidant activities in cells and *C. elegans*. For example, thistle extract exhibited the protective effects against ROS in PC 12 cells and also increased the lifespan of *C. elegans* under the oxidative stress [25]. Pasteurized orange juice rich in carotenoids promotes survival of *C. elegans* to oxidative stress [26]. Notability, RCJ treatment provided better results on stress tolerance in cells and *C. elegans* compared to GCJ. These benefits could be explained based on the higher total phenolics, ascorbic acid, glucosinolates, and anthocyanins content of RCJ compared with that of GCJ.

In *C. elegans*, RCJ increased aging-related healthspan and lifespan. Both RCJ and GCJ treatment decreased the autofluorescence accumulation and brood size, increased the body length, but did not reduce ROS level. These results showed a similar trend between RCJ and GCJ treatment, which might not attribute to the different amount of total phenolics, ascorbic acid, glucosinolates, and anthocyanins. According to “free radical theory of aging”, antioxidants supplementation was expected to be beneficial to lifespan. However, many studies demonstrated that antioxidant effect was unlikely to be the main factor for lifespan extension [27,28]. Phytochemicals could target signal pathways and molecules to removing cellular damage [29]. While high ROS levels are recognized to cause cellular damage and to promote aging, comparably low amounts of ROS may exert positive effects on the biological process by inducing some adaptive response, which is called “mitochondrial hormesis” [30].

Autofluorescent materials have accumulated over time and were often used as a marker of aging in *C. elegans*. Our results revealed that RCJ treatment significantly decreased autofluorescence, while GCJ treatment had no impact on the accumulation of autofluorescent materials in worms. Blue fluorescence was widely used in analysis of autofluorescent materials [10]. Notably, a recent study reported that red autofluorescence was more appropriate to use to characterize aging than blue and green autofluorescence [31]. Endogenous ROS was determined by the H_2_DCF-DA assay. RCJ and GCJ were not able to provoke any changes in ROS level. An explanation was that the antioxidant activity of RCJ was not sufficient to reduce the accumulation of ROS in worms. Another factor may be that the H_2_DCFDA assay relied on ingestion and membrane permeability to enter into the worm; the different efficiencies of H_2_DCFDA cells may lead to misinterpretation [32]. Lin, Zhang, Xiao, Zhong, Kuang, Cao and Chen [10] showed a similar result that carnosic acid treatment improved the survival rate under oxidative stress, but did not reduce ROS accumulation.

Body length for worms treated with RCJ and GCJ was significantly increased (*p* < 0.05), suggesting that RCJ and GCJ promoted the growth of *C. elegans*. Similarly, withanolide A treatment increased the body length and width on day 2, 5, and 10 of adulthood [33]. Gallic acid treatment significantly increased the body length of worms [34]. Brood size was decreased by RCJ and GCJ treatment, indicating that RCJ and GCJ impaired the fertility rate. According to “disposable soma” theory, the reduced reproduction can divert the energy to maintenance of the adult [35]. Interestingly, withanolide A and gallic acid also decreased brood size, which exhibited similar trends with RCJ [33,34].

In the current study, RCJ treatment significantly extended lifespan in a dose-dependent manner, and mean lifespan of wild-type worms increased from 19.57 to 25.08 days in the presence of 5% RCJ. GCJ treatments were not able to prolong the lifespan of wild-type worms. In view of these findings, we speculate that the content of phytochemicals and some specific phytochemicals might play a key a role in lifespan extension. Previous studies have shown that anthocyanins can provide a variety of health benefits. For example, Chen et al. [36] showed that anthocyanin-rich extract of purple wheat extended the mean lifespan of wild-type *C. elegans* by 10.5% and the lifespan extension depended on the transcription factor DAF-16. Likewise, mulberry anthocyanin extract extended the lifespan of *C. elegans* [37]. Peixoto et al. [38] showed that anthocyanin-rich extract of Acai protected worms against oxidative stress, but no lifespan extension effect was observed.

IIS pathway is one of the most potent facilitators of longevity in *C. elegans*, which includes the DAF-2 transmembrane receptor and DAF-16/FOXO transcription factor [39]. The inactivation of *daf-2* leads to the activation of *daf-16*, which prolongs the lifespan of *C. elegans* [40]. RCJ significantly up-regulated *daf-2* mRNA level, but did not alter the mRNA level of *daf-16* and *sod-3* with statistically significant differences. Furthermore, RCJ significantly (*p* < 0.05 by the log-rank test) prolonged the lifespan of the *daf-16(mgDf50)* mutants, suggesting that IIS pathway was not a direct target of RCJ. Additionally, RCJ did not extend the lifespan of *sir-2.1(ok434)* mutant. Sirtuin signaling might be part of the mechanism that increased the longevity induced by RCJ (Figure 5E). SIR-2.1, a member of the Sir-2 family of NAD^+^-dependent protein deacetylases, extended the lifespan via the transcription factor *daf-16* [41], whereas Wang and Tissenbaum [42] also reported that *sir-2.1* and *daf-16* might have a distinct function, since *sir-2.1* exhibited lifespan extension by caloric restriction pathway. Similarly, treatment of *C. elegans* with resveratrol extended lifespan dependent upon *sir-2.1*, but independent of *daf-16* [11].

The transcription factor HSF-1 controls the inducible transcription of genes encoding heat shock proteins (HSPs), which acts by counteracting the misfolded proteins in cytoplasm and nucleus [43]. RCJ did not significantly affect the *hsf-1* gene expression, but increased the expression of *hsf-1* target *hsp-16.1* and *hsp-16.2*. RCJ might act through inducing the translocation of HSF-1 to the nucleus to modulate adaptive responses to environmental changes (Figure 5E). Increased heat shock proteins (HSPs) are known to have enhanced stress resistance in *C. elegans*, which are consistent with the increased resistance to oxidative and heat stress [44].

In *C. elegans*, the resistance to hyperosmotic stress requires several proteins of the CaMKII pathway, in which OSR-1 is coupled to SEK-1/mitogen-activated protein (MAP) kinase through UNC-43/CaMKII [45]. RCJ did not prolong the lifespan of the *unc-43(n498n1186)* mutants, suggesting that *unc-43* was necessary for RCJ-mediated lifespan extension. RCJ down-regulated expression of *osr-1* but not alter the expression of *sek-1*. These findings indicated that the action of RCJ was likely mediated through CaMKII pathways (Figure 5E). Similarly, *osr-1*, but not *sek-1*, was required for cranberry-induced longevity [46]. Wilson, Shukitt-Hale, Kalt, Ingram, Joseph and Wolkow [17] found that blueberry polyphenol-induced longevity required the presence of the CaMKII pathway that mediated osmotic stress resistance.

The results of this study have several limitations. First, blinding was suggested for survival studies in worms. A randomized trial without blinding might show larger effects than a blinded study [47]. Blinding of manual measurement assay was found to increase consistency between operators and replication. Additionally, the timing of administration was important in relation to the phytochemical effects in worms [48]. Guha, Natarajan, Murbach, Dinh, Wilson, Cao, Zou and Dong [30] reported that early start intervention (supplementation started from L1 stage) with cranberry extract significantly promoted healthspan and led to more prominent benefits as compared to late-start interventions (supplementation started from L4 stage). In this study, RCJ and GCJ were administered to worms from the beginning of L1 or L4 larval stage, it was not clear whether RCJ exhibited similar effects in the same larval developmental stage or in aged worms.

## 5. Conclusions

Our study demonstrated that RCJ rather than GCJ exhibited protected effects in both oxidative stressed caco-2 cells and *C. elegans* and led to a life-prolonging effect under standard laboratory conditions. RCJ could target multiple longevity mechanisms including Sirtuin signaling, HSF-1 pathway, and CaMKII pathway. These results might be attributed to higher total phenolics, ascorbic acid, glucosinolates, and anthocyanins content of RCJ compared with that of GCJ.

## Figures and Tables

**Table 1:** Total phenolics, ascorbic acid, glucosinolates, and anthocyanins content in red cabbage juice (RCJ) and green cabbage juice (GCJ) (mg/100 mL of juice).

	RCJ	GCJ
Phenolics ^a^	29.95 ± 0.94	17.20 ± 0.15 *
Ascorbic acid	30.55 ± 0.03	21.68 ± 0.17 *
Glucosinolates ^b^	70.21 ± 3.64	59.66 ± 1.04 *
Anthocyanins ^c^	33.87 ± 0.60	none

Values are represented as means ± SD (*n* = 3); * *p* < 0.05 vs. RCJ. ^a^ Total phenolics were calculated as gallic acid equivalents (mg GAE/100 mL of juice). ^b^ Total glucosinolates were calculated as mg sinigrin equivalent/100 mL of juice. ^c^ Total anthocyanins were calculated as cyanidin 3-glucoside (C3G) equivalents (mg C3G/100 mL of juice).

**Table 2:** Identification of red cabbage juice anthocyanins by UPLC-MS analysis.

Peak ^a^	Retention Time (min)	[M]^+^ (*m*/*z*)	Fragments (*m*/*z*)	Tentative Identification
1	4.49	773.21	287.05	Cyanidin 3-diglucoside-5-glucoside
2	6.60	1081.3	919.25/502.86/449.11/287.05	Cyanidin 3-(caffeoyl)(*p*-coumaroyl)-diglucoside-5-glucoside
3	6.77	1111.31	1111.31/949.26/606.18/449.11/287.05	Cyanidin 3-(caffeoyl)(feruloyl)-diglucoside-5-glucoside
4	8.13	919.25	919.25/757/287.05	Cyanidin 3-(*p*-coumaroyl)-diglucoside-5-glucoside
5	8.31	949.26	949.26/787.2/449.11/287.05	Cyanidin 3-(feruloyl)-diglucoside-5-glucoside
6	8.36	979.27	979.27/817.22/449.11/287.05	Cyanidin 3-(sinapoyl)-diglucoside-5-glucoside
7	9.15	1185.306	1185.33/1023.28/993.27/569.31/449.11/287.05	Cyanidin 3-(sinapoyl)-diglucoside-5-(sinapoyl)-glucoside

^a^ Peak numbers are the same as the peaks shown in the chromatograms in Figure 1.

**Table 3:** Statistical analysis of the lifespan of *C. elegans*.

Condition	Treatment	Number	Mean Lifespan (Days) ^a^	% of Control
N2	0	116	19.57 ± 0.45	
	1% RCJ	150	20.63 ± 0.35	5.40
	2% RCJ	159	21.60 ± 0.32 *	10.37
	3% RCJ	148	22.12 ± 0.29 *	13.04
	5% RCJ	157	25.08 ± 0.48 *	28.18
	1% GCJ	196	19.60 ± 0.45	0.16
	2% GCJ	208	20.09 ± 0.26	2.65
	3% GCJ	227	20.26 ± 0.26	3.51
	5% GCJ	155	20.65 ± 0.48	5.50
GR1370	0	102	15.98 ± 0.27	
	5% RCJ	105	17.41 ± 0.11 *	8.95
VC199	0	88	18.82 ± 0.40	
	5% RCJ	70	18.88 ± 0.72	0.32
MT2605	0	77	20.47 ± 0.62	
	5% RCJ	76	21.50 ± 0.24	5.03

^a^ Mean ± SEM. * *p* < 0.05 vs. control, log-rank test. RCJ: red cabbage juice, GCJ: green cabbage juice.

## Data Availability

All data included in this study are available upon reasonable request by contacting the corresponding author.

## Supplementary Materials

The following are available online at https://www.mdpi.com/article/10.3390/antiox10060930/s1, Table S1: Quantitative PCR primers.
