# Supplementary material for: Red Cabbage Rather Than Green Cabbage Increases Stress Resistance and Extends the Lifespan of Caenorhabditis elegans
Source: Antioxidants (Basel). 2021 Jun 8;10(6):930. doi: 10.3390/antiox10060930 (PMC8228718; doi:10.3390/antiox10060930)
Supplement: Supplementary file 1 [file antioxidants-10-00930-s001.zip › antioxidants-1232429-SI.pdf]

antioxidants

Supplementary Material

Red Cabbage Rather Than Green Cabbage Increases Stress Resistance and Extends the Lifespan of *Caenorhabditis elegans*

Nan Zhang, Shunshan Jiao and Pu Jing*

Shanghai Food Safety and Engineering Technology Research Center, Key Lab of Urban Agriculture Ministry of Agriculture, School of Agriculture & Biology, Shanghai Jiao Tong University, Shanghai 200240, China; nzhang@sjtu.edu.cn (N.Z.); sjiao@sjtu.edu.cn (S.J.)

* Correspondence: pjing@sjtu.edu.cn; Tel.: +86-21-3420-7074

Table S1. Quantitative PCR primers.

Gene	Forward	Reverse	Genes accession number	Annealing temperature	Efficiency
daf-2	GGATAAAGGCGAATC AAAGTGTC	CGATACACTTTCCCTT GTGATAGAC	AF012437.1	60	101.5
daf-16	TTTCCGTCCCCGAACT CAA	ATTCGCCAACCCATG ATGG	AF032112.1	60	98.4
sod-3	CCAACCAGCGCTGAA ATTCAATGG	GGAACCGAAGTCGCG CTTAATAGT	NM_078363.9	60	95.8
hsf-1	TTGACGACGACAAGC TTCCAGT	AAAGCTTGCACCAGA ATCATCCC	AY559748.1	60	102.3
hsp-16.1	CTGAATCTTCTGAGAT TGTTAAC	TTTGTTCAACGGGCGC TTGC	NM_072953.5	60	97.2
hsp-16.2	CGTCGAAGAGAAATC TGCTGAA	TGCAGCGAACAATAC TGTAATTTATG	NM_001392482.1	60	97.1
sek-1	TTATGGAGCAGGCAA GAAATG	AAAGACTTGTTCGCC ATTCG	AB024087.1	60	98.2
osr-1	AGCATCATGCCACCT ACGTGA	CACCACCATTGAATTT CAGCG	AY360470.1	60	99.4
act-1	CCAGGAATTGCTGAT CGTATGCAGAA	TGGAGAGGGAAGCGA GGATAGA	NM_073418.9	60	103.5
